# Supplementary material for: High Prevalence of Plasmid-Mediated Quinolone Resistance among ESBL/AmpC-Producing Enterobacterales from Free-Living Birds in Poland
Source: Int J Mol Sci. 2023 Aug 15;24(16):12804. doi: 10.3390/ijms241612804 (PMC10454011; doi:10.3390/ijms241612804)
Supplement: Supplementary file 1 [file ijms-24-12804-s001.zip › ijms-2550592-supplementary.pdf]

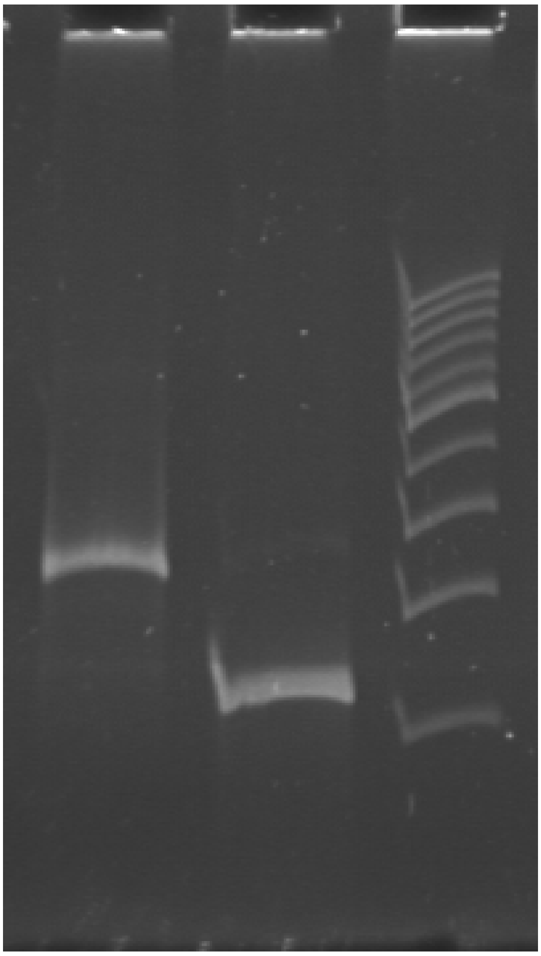

Figure S1. Gel electrophoresis showing an example of PCR product digestion by restriction enzyme Csp45I. Lane M – 100 bp size marker. Lane P1 - undigested PCR product (263 bp); lane P2 - PCR product cut into two fragments with Csp45I (134 bp and 129 bp).
